# Tinnitus patient challenges and frustrations with current treatments

**DOI:** 10.1016/j.clinsp.2026.100955

**Published:** 2026-04-22

**Authors:** Anna Carolina Marques Perrella de Barros, Richard S. Tyler

**Affiliations:** a Member of the Tinnitus and Sound Intolerance Group, Universidade Federal de São Paulo (UNIFESP), São Paulo, SP, Brazil; b Department of Otolaryngology and Speech Pathology & Audiology University of Iowa, Iowa City, Iowa, USA

## Introduction

Many with tinnitus experience severe distress and have difficulty finding help.^1^^,^^2^ Difficult issues patients often face when seeking help for tinnitus include searching for a magic pill, navigating apps and internet sites, lack of care in many hospitals and clinics, and the range of hearing aids and sound therapies available. There are many subtypes of tinnitus, each requiring a different counseling approach. Some patients have hyperacusis, with a different set of severe challenges. The lack of reimbursement for tinnitus providers is yet another major obstacle.

Understanding the needs and expectations of tinnitus patients remains challenging for some healthcare providers. This article aims to prepare clinicians to support individuals with tinnitus. Building on this, we discuss the issues in key areas that impact tinnitus care. Additionally, we suggest several approaches to enhance professional capacity.

## Searching for the magic pill

Many with tinnitus initially expect (and hope) for a pill that will eliminate their tinnitus.^3^ Some will spend much time searching online and ask their healthcare providers for a pill. When they realize there is no pill, they can be very depressed.

We, as clinicians, can share that many have tinnitus and that their concerns are reasonable. Self-help books can be provided.^4^ You can tell them you offer clinical services, and/or referrals can be made to others who provide tinnitus care.

## Seeking help on the internet and apps

Of course, many tinnitus sufferers will seek help online and through apps.^4^^,^^5^ Caution is needed due to unrealistic marketing attempts.^4^ There are many important individual differences among patients related to the problems they experience and their severity. Sometimes marketing confuses patients.

However, some websites might be helpful. There are healthcare professionals committed to providing quality information, with responsible, ethical, and evidence-based clinical services.^5^

A variety of tinnitus associations and self-help groups can be a useful starting place. This helps to learn that they are not alone, that there are helpful providers, and that several treatment options are available.^4^

Caution is needed because of important individual needs.^3^^,^^6^ Those with distressing tinnitus should seek professional help. Having specialized professional guidance is more secure than adopting strategies on one's own.

## Seeking help at hospitals and clinics

Many patients initially seek help from their assigned healthcare provider. Many patients will not be aware of the tinnitus network support. Certainly, physicians can rule out other health issues and make referrals to audiologists. Psychologists can provide emotional support. Unfortunately, most healthcare providers do not get training on helping tinnitus patients with their different and sometimes severe reactions.^2^^,^^3^ It is important for healthcare professionals to work together and make referrals to other providers when needed.

## Hearing aids and sound therapy

Many patients have pure-tone threshold losses and tinnitus that interfere with hearing, and many will benefit from hearing aids and audiological counseling.^1^ However, the pure-tone threshold loss might not be severe enough for many audiologists to recommend and fit hearing aids. Sound therapy alone can still be helpful.^2^^,^^6^ Many hearing aids have sound generators that can be adjusted for tinnitus. Sound therapy includes several options, including broadband noise, waves, background music, and more. Having some low-level background sound can reduce the prominence of the tinnitus. Low levels are important, so the sound does not interfere with hearing.^6^

## Hyperacusis

Some patients with tinnitus also experience loudness, annoyance, fear, or pain hyperacusis.^7^ Hyperacusis patients have complaints about the sounds and situations that sounds are troublesome. Symptoms vary individually.^7^ Its presence should be determined initially, as it can be more disruptive than the tinnitus and might need to be treated before the tinnitus.^7^

## Subtypes of tinnitus

For therapy, defining subtypes can be an essential step for delivering effective approaches.^2^^,^^3^ Generally, Thoughts and Emotions, Hearing, Sleep, and Concentration are the different difficulties faced by tinnitus sufferers.^6^ The Tinnitus Primary Functions Questionnaire helps clinicians and patients identify the problems that they experience and should be addressed by counseling.^6^

## Thoughts and emotions

Some patients will be very distressed. They might think there is no treatment.^1^ Tinnitus can be difficult to explain to others. Thoughts and emotions are influenced by tinnitus and modulate reactions to tinnitus.^6^ Connecting with others with tinnitus (for example, in a group counseling session) can be an important first step (for the tinnitus sufferer and, in some cases, their partner).^6^^,^^8^

## Hearing

Most of the tinnitus patients will have some difficulty hearing. This will likely include both the sensorineural hearing loss and hearing difficulties from the tinnitus.^6^ A threshold of 10 dB HL might actually represent a pure-tone threshold loss for some.^9^ In addition, tinnitus can interfere with perception. Many will benefit from hearing aids and aural rehabilitation.^1^^,^^3^

## Sleep

Tinnitus at bedtime can be distracting, affect sleep quality, and lead to sleep disturbances.^3^^,^^6^ Audiologists can provide useful strategies to help patients sleep.^6^ A simple approach is to have a low-level, comfortable sound in the bedroom that can be ignored, all night long.^4^^,^^6^

## Concentration

Attentional problems caused by tinnitus are often not appreciated by clinicians. Tinnitus affects concentration, and distracting attention from tinnitus can be extremely challenging.^6^ Counseling focused on controlling attention can help.^4^^,^^6^ Having a low-level background sound can reduce the prominence of the tinnitus and can also help. Tinnitus Activities Treatment includes training with attentional tasks varying in complexity.^6^

## Reimbursement

Much healthcare is covered by the American Medical Association. This does not cover audiological care focused on tinnitus.^10^ Some healthcare plans do. Many tinnitus sufferers want help and are willing to pay for professional services.

## Conclusions

Many patients have tinnitus and are very distressed. Several approaches can contribute to effective management. Supported patients can seek help with confidence and improve their quality of life. We can help them! We need to understand their individual problems, what they have tried, and what support they have. It can be very rewarding for healthcare providers to offer tinnitus care and help sufferers!

## Generative AI use statement

No generative AI tools were used in the preparation of this work. All writing, analysis, and editing were completed by the authors.

## Ethics approval statement

This manuscript used data from publicly available sources and did not involve human participants or identifiable personal data; therefore, ethical approval was not required.

## Data availability

No new data were created or analyzed in this study. Therefore, data sharing is not applicable.

## Conflicts of interest

The authors declare no conflicts of interest.
